# Rotation-Invariant Features for Multi-Oriented Text Detection in Natural Images

**DOI:** 10.1371/journal.pone.0070173

**Published:** 2013-08-05

**Authors:** Cong Yao, Xin Zhang, Xiang Bai, Wenyu Liu, Yi Ma, Zhuowen Tu

**Affiliations:** 1 Department of Electronics and Information Engineering, Huazhong University of Science and Technology, Wuhan, China; 2 Department of Computer Science and Technology, Tsinghua University, Beijing, China; 3 Department of Electronics and Information Engineering, Huazhong University of Science and Technology, Wuhan, China; 4 Department of Electronics and Information Engineering, Huazhong University of Science and Technology, Wuhan, China; 5 Microsoft Research Asia, Beijing, China; 6 University of California Los Angeles, Los Angeles, California, United States of America; University of Warwick, United Kingdom

## Abstract

Texts in natural scenes carry rich semantic information, which can be used to assist a wide range of applications, such as object recognition, image/video retrieval, mapping/navigation, and human computer interaction. However, most existing systems are designed to detect and recognize horizontal (or near-horizontal) texts. Due to the increasing popularity of mobile-computing devices and applications, detecting texts of varying orientations from natural images under less controlled conditions has become an important but challenging task. In this paper, we propose a new algorithm to detect texts of varying orientations. Our algorithm is based on a two-level classification scheme and two sets of features specially designed for capturing the intrinsic characteristics of texts. To better evaluate the proposed method and compare it with the competing algorithms, we generate a comprehensive dataset with various types of texts in diverse real-world scenes. We also propose a new evaluation protocol, which is more suitable for benchmarking algorithms for detecting texts in varying orientations. Experiments on benchmark datasets demonstrate that our system compares favorably with the state-of-the-art algorithms when handling horizontal texts and achieves significantly enhanced performance on variant texts in complex natural scenes.

## Introduction

Texts in a natural scene directly carry critical high-level semantic information. Their existence is also ubiquitous in urban environments, e.g. traffic signs, billboards, business name cards, and license plates. Effective text detection and recognition systems have been very useful in a variety of applications such as robot navigation [1], image search [2], and human computer interaction [3]. The popularity of smart phones and ubiquitous computing devices have also made the acquisition and transmission of text data increasingly convenient and efficient. Thus, automatically detecting and recognizing texts from casually captured images has become an ever important task in computer vision.

In this paper, we tackle the problem of text detection in natural images, which remains a challenging task although it has been extensively studied in the past decades [4]–[19]. The difficulty of automatic text detection mainly stems from two aspects: (1) diversity of text appearances and (2) complexity of cluttered backgrounds. On one hand, texts, unlike conventional objects (e.g. cars and horses), typically consist of a large number of different instances and they exhibit significant variations in shapes and appearances: different texts may have different sizes, colors, fonts, languages, and orientations, even within the same scene. On the other hand, many other man-made objects (such as windows and railings) in the scene often bear a great deal of similarity to texts. Sometimes even natural objects (such as grasses and leaves) may happen to distribute in a similar way as a sequence of characters. Such ambiguities have made reliable text detection in natural images a challenging task.

**Figure 1:**
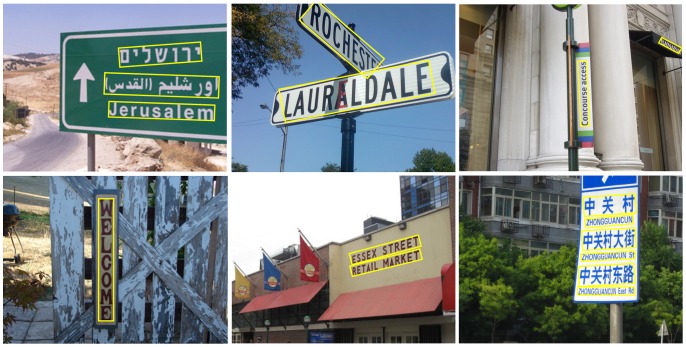
Detected texts in natural images by the proposed algorithm.

In the literature, most of the existing methods [6], [9], [20] have focused on detecting horizontal or near-horizontal texts, as we will see in a survey of related work. Obviously, the requirement of being horizontal severely limits the applicability of those methods in scenarios where images are taken casually with a mobile device. Detecting texts with varying orientations in complex natural scenes remains a challenge for most practical text detection and recognition systems [21], [22]. In this work, we aim to build an effective and efficient system for detecting multi-oriented texts in complex natural scenes (see Fig. 1).

Most conventional text detection methods rely on features that are primarily designed for horizontal texts (such as those used in [7], [13], [19]). Thus, when such methods are applied to images that contain multi-oriented texts, their performance usually drops drastically. To remedy this situation, we introduce two additional sets of rotation-invariant features for text detection. To further reduce false positives produced by only using such low-level features, we have also designed a two-level classification scheme that can effectively discriminate texts from non-texts. Hence, by combining the strength of rotation-invariant features and well trained text classifiers, our system is able to effectively detect multi-oriented texts with very few false positives.

The proposed method is mostly bottom-up (data-driven) but with additional prior knowledge about texts imposed in a top-down fashion. Pixels are first grouped into connected components, corresponding to strokes or characters; connected components are then linked together to form chains, corresponding to words or sentences. The connected components and chains are verified by the orientation-invariant features and discriminative classifiers. With this strategy, our method is able to combine the strength of both prior knowledge about texts (such as uniform stroke width) and automatically learned classifiers from labeled training data. In this way, we can strike a good balance between systematic design and machine learning, which is shown to be advantageous over either heavy black-box learning [7] or purely heuristic design [9].

To evaluate the effectiveness of our system, we have conducted extensive experiments on both conventional benchmarks and some new (more extensive and challenging) datasets. Compared with the state-of-the-art text detection algorithms, our system performs competitively in the conventional setting of horizontal texts. We have also tested our system on a challenging dataset of 500 natural images containing texts of various orientations in complex backgrounds. On this dataset, our system works significantly better than the existing systems, with an F-measure about 0.6, more than twice that of the closest competitor.

We have presented a preliminary version of our work in [23]. This paper extends that article with the following contributions: (1) some steps of the algorithm are improved. Specifically, the case of detecting single characters, which is heavily neglected by existing methods, is discussed; (2) further evaluations, including text detection experiments on the dataset of the latest ICDAR robust reading competition (ICDAR 2011) and on texts of different languages, are conducted; (3) an end-to-end multi-oriented scene text recognition system, integrating the proposed text detection algorithm with an off-the-shelf OCR engine, is introduced; (4) the proposed evaluation protocol is detailed; (5) more technical details of the proposed method are presented and (6) comprehensive discussions and analyses are given.

### Related Work

There have been a large number of systems dealing with text detection in natural images and videos [4]–[18], [24]–[28]. Comprehensive surveys can be found in [29], [30]. Existing approaches to text detection can be roughly divided into three categories: texture-based, component-based, and hybrid methods.

#### Three categories of existing approaches

**Texture-based methods** (e.g. [6], [7], [24]) treat text as a special type of texture and make use of its textural properties, such as local intensities, spatial variance, filter responses, and wavelet coefficients. Generally, these methods are computation demanding as all locations and scales are exhaustively scanned. Moreover, these algorithms mostly only detect horizontal texts.

In an early work, Zhong *et al.* [31] proposed a method for text localization in color images. Horizontal spatial variance was used to roughly localize texts and color segmentation was performed within the localized areas to extract text components. The system of Wu *et al.* [32] adopted a set of Gaussian derivatives to segment texts. Rectangular boxes surrounding the corresponding text strings were formed, based on certain heuristic rules on text strings, such as height similarity, spacing and alignment. The above steps were applied to an image pyramid and the results were fused to make final detections. Li *et al.* [33] presented a system for detecting and tracking texts in digital video. In this system, the mean and the second- and third-order central moments of wavelet decomposition responses are used as local features. Zhong *et al.* [34] proposed to localize candidate caption text regions directly in the discrete cosine transform (DCT) compressed domain using the intensity variation information encoded in the DCT domain. The method proposed by Gllavata *et al.* [24] utilized the distribution of high-frequency wavelet coefficients to statistically characterize text and non-text areas.

Different from the methods surveyed above, in which filter responses or transform domain coefficients are used as features, the algorithm of Kim *et al.* [6] relies merely on intensities of raw pixels. A Support Vector Machine (SVM) classifier is trained to generate probability maps, in which the positions and extents of texts are searched using adaptive mean shift. Lienhart and Wernicke [35] used complex-valued edge orientation maps computed from the original RGB image as features and trained neural network to distinguish between text and non-text patterns.

The method of Weinman *et al.* [36] used a rich representation that captures important relationships between responses to different scale- and orientation-selective filters. To improve the performance, conditional random field (CRF) was used to exploit the dependencies between neighboring image region labels. Based on the observation that areas with high edge density indicate text regions, text detection in [37] was carried out in a sequential multi-resolution paradigm.

To speed up text detection, Chen *et al.* [7] proposed an efficient text detector, which is a cascade Adaboost classifier. The weak classifiers are trained on a set of informative features, including mean and variance of intensity, horizontal and vertical derivatives, and histograms of intensity gradient. Recently, Wang *et al.* [10] present a method for spotting words in natural images. They first perform character detection for every letter in an alphabet and then evaluate the configuration scores for the words in a specified list to pick out the most probable one.

**Component-based methods** (e.g. [4], [9], [14], [38]) first extract candidate text components through various ways (e.g. color reduction [4], [14] and Maximally Stable Extremal Region detection [11], [25]) and then eliminate non-text components using heuristic rules or trained classifier, based on geometry and appearance properties. Component-based methods are usually more efficient than texture-based methods because the number of candidate components is relatively small. These methods are more robust to the variations of texts, such as changes of font, scale and orientation. Moreover, the detected text components can be directly used for character recognition. Due to these advantages, recent progresses in text detection and recognition in natural images have been largely advanced by this category of methods [9], [11], [14], [18], [38]–[40].

In [4], color reduction and multi-valued image decomposition are performed to partition the input image into multiple foreground components. Connected component analysis is applied to these foreground components, followed by a text identification module, to filter out non-text components.

The great success of sparse representation in face recognition [41] and image denoising [42] has inspired numerous researchers in the community. The authors of [43] and [12] apply classification procedure to candidate text components, using learned discriminative dictionaries.

The MSER-based methods [11], [18], [25], [40] have attracted much attention from the community, because of the excellent characteristics of MSERs (Maximally Stable Extremal Regions) [44]. MSERs can be computed efficiently (near linear complexity) and are robust to noise and affine transformation. In [11], MSERs are detected and taken as candidate text components. Neumann *et al.* [40] modified the original MSER algorithm to take region topology into consideration, leading to superior detection performance. Chen *et al.* [25] also proposed an extension to MSER, in which the boundaries of MSERs are enhanced via edge detection, to cope with image blur. Recently, Neumann *et al.* [18] further extend the work of [11], [40] to achieve real-time text detection and recognition.

Epshtein *et al.* [9] proposed a novel image operator, called Stroke Width Transform (SWT), which transforms the image data from containing color values per pixel to containing the most likely stroke width. Based on SWT and a set of heuristic rules, this algorithm can reliably detect horizontal texts.

While most existing algorithms are designed for horizontal or near-horizontal texts, Yi *et al.* [14] and Shivakumara *et al.* [16] consider the problem of detecting multi-oriented texts in images or video frames. After extracting candidate components using gradient and color based partition, the line grouping strategy in [14] aggregates the components into text strings. The text strings can be in any direction. However, the method of [14] relies on a large set of manually defined rules and thresholds. In [16], candidate text component clusters are identified by *K*-means clustering in the Fourier-Laplacian domain. The component clusters are divided into separate components using skeletonization. Even though this method can handle multi-oriented texts, it only detects text blocks, rather than characters, words or sentences.

Finally, **hybrid methods** (e.g. [13], [45]) are a mixture of texture-based and component-based methods. In [45], edge pixels of all possible text regions are extracted, using an elaborate edge detection method; the gradient and geometrical properties of region contours are verified to generate candidate text regions, followed by a texture analysis procedure to distinguish true text regions from non-text regions. Unlike [45], the hybrid method proposed by Pan *et al.* [13] extracts candidate components from probability maps at multiple scales. The probability maps are estimated by a classifier, which is trained using a set of texture features (HOG features [46]) computed in predefined patterns. Like most other algorithms, these two methods only detect horizontal texts.

### Our Strategy

We have drawn two observations about the current text detection algorithms: (1) methods that are purely based on learning (nearly black-box) [7] by training classifiers on a large amount of data can reach certain but limited level of success (system [7] obtained from the authors produces reasonable results on horizontal English texts but has poor performance in general cases); (2) systems that are based on smart features, such as Stroke Width Transform (SWT) [9], are robust to variations of texts but they involve a lot of tuning and are still far from producing all satisfactory results, especially for non-horizontal texts.

In this paper, we adopt SWT and also design various new features that are intrinsic to texts and robust to variations (such as rotation and scale change); a two-level classification scheme is devised to moderately utilize training to remove sensitive parameter tuning by hand. We observe significant improvement over the existing approaches in dealing with real-world scenes.

Though widely used in the community, the ICDAR datasets [47]–[49] only contain horizontal English texts. In [14], a dataset with texts of different directions is released, but it includes only 89 images without enough diversity in the texts and backgrounds. Here we collect a new dataset with 500 images of indoor and outdoor scenes. In addition, the evaluation methods used in [50] and the ICDAR competitions [47]–[49] are mainly designed for horizontal texts. Hence, we propose a new protocol that is more suitable for assessing algorithms developed for multi-oriented texts.

### Proposed Approach

**Figure 2:**
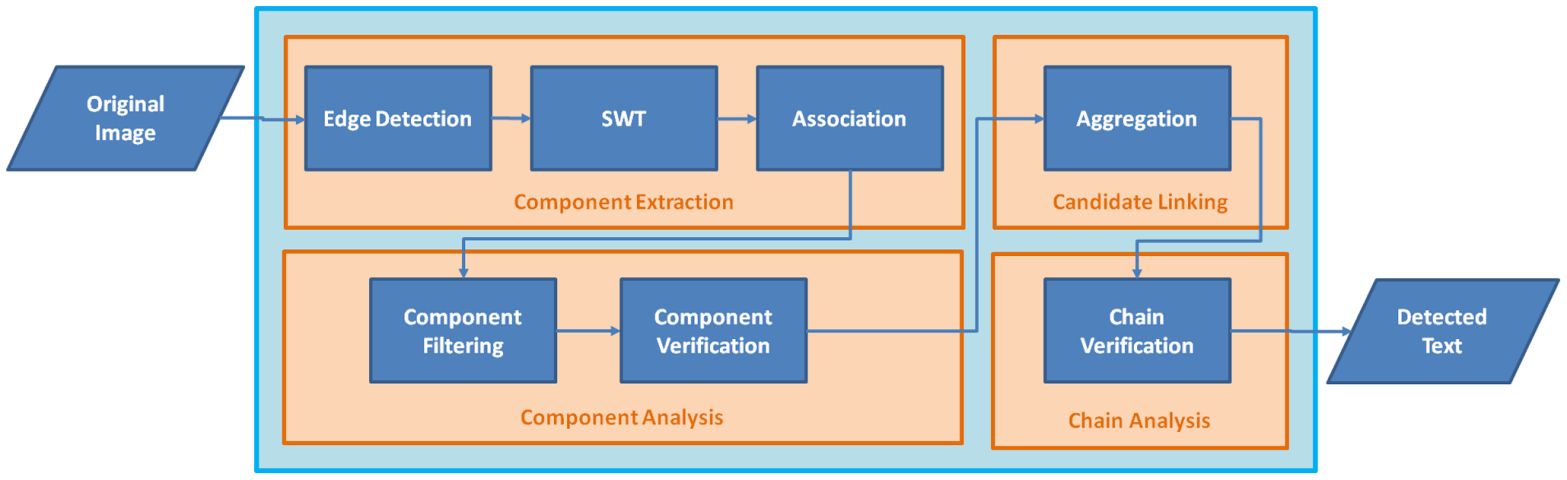
Pipeline of the proposed approach.

The proposed algorithm consists of four stages: (1) component extraction, (2) component analysis, (3) candidate linking, and (4) chain analysis, which can be further categorized into two procedures, bottom-up grouping and top-down pruning, as shown in Fig. 2. In the bottom-up grouping procedure, pixels are first grouped into connected components and later these connected components are aggregated to form chains; in the top-down pruning procedure non-text components and chains are successively identified and eliminated. The two procedures are applied alternately.

#### Component extraction

At this stage, edge detection is performed on the original image and the edge map is fed to the SWT [9] module to produce an SWT image. Neighboring pixels in the SWT image are grouped together recursively to form connected components using a simple association rule.

#### Component analysis

Many components extracted at the component extraction stage are not parts of texts. The component analysis stage is aimed to identify and filter out those non-text components. First, the components are filtered using a set of heuristic rules that can distinguish between obvious spurious text regions and true text regions. Next, a component level classifier is applied to prune the non-text components that are hard for the simple filter.

#### Candidate linking

The remaining components are taken as character candidates. In fact, components do not necessarily correspond to characters, because a single character in some languages may consist of several strokes; however, we still call them characters (or character candidates) hereafter for simplicity. The first step of the candidate linking stage is to link the character candidates into pairs. Two adjacent candidates are grouped into a pair if they have similar geometric properties and colors. At the next step, the candidate pairs are aggregated into chains in a recursive manner.

#### Chain analysis

At the chain analysis stage, the chains determined at the former stage are verified by a chain level classifier. The chains with low classification scores (probabilities) are discarded. The chains may be in any direction, so a candidate might belong to multiple chains; the interpretation step is aimed to dispel this ambiguity. The chains that pass this stage are the final detected texts.

The remainder of this paper is organized as follows. Section **Methodology** presents the details of the proposed method, including the algorithm pipeline and the two sets of features. Section **Dataset and Evaluation Protocol** introduces the proposed dataset and evaluation protocol. The experimental results and discussions are given in Section **Experiments and Discussions**. Section **Conclusions** concludes the paper and points out potential directions for future research.

## Methodology

In this section, we present the details of the proposed algorithm. Specifically, the pipeline of the algorithm will be presented in Section **Algorithm Pipeline** and the details of the features will be described in Section **Feature Design**.

### Algorithm Pipeline

#### Component extraction

To extract connected components from the image, SWT [9] is adopted for its effectiveness and efficiency. SWT is an image operator which computes per pixel width of the most likely stroke containing the pixel. It provides a way to discover connected components from edge map directly, which makes it unnecessary to consider the factors of scale and direction. See [9] for details.

SWT runs on edge map, so we use Canny edge detector [51] to produce an edge map (Fig. 3 (b) of [23]) from the original image (Fig. 3 (a) of [23]). The resulting SWT image is shown in Fig. 3 (c) of [23].

**Figure 3:**
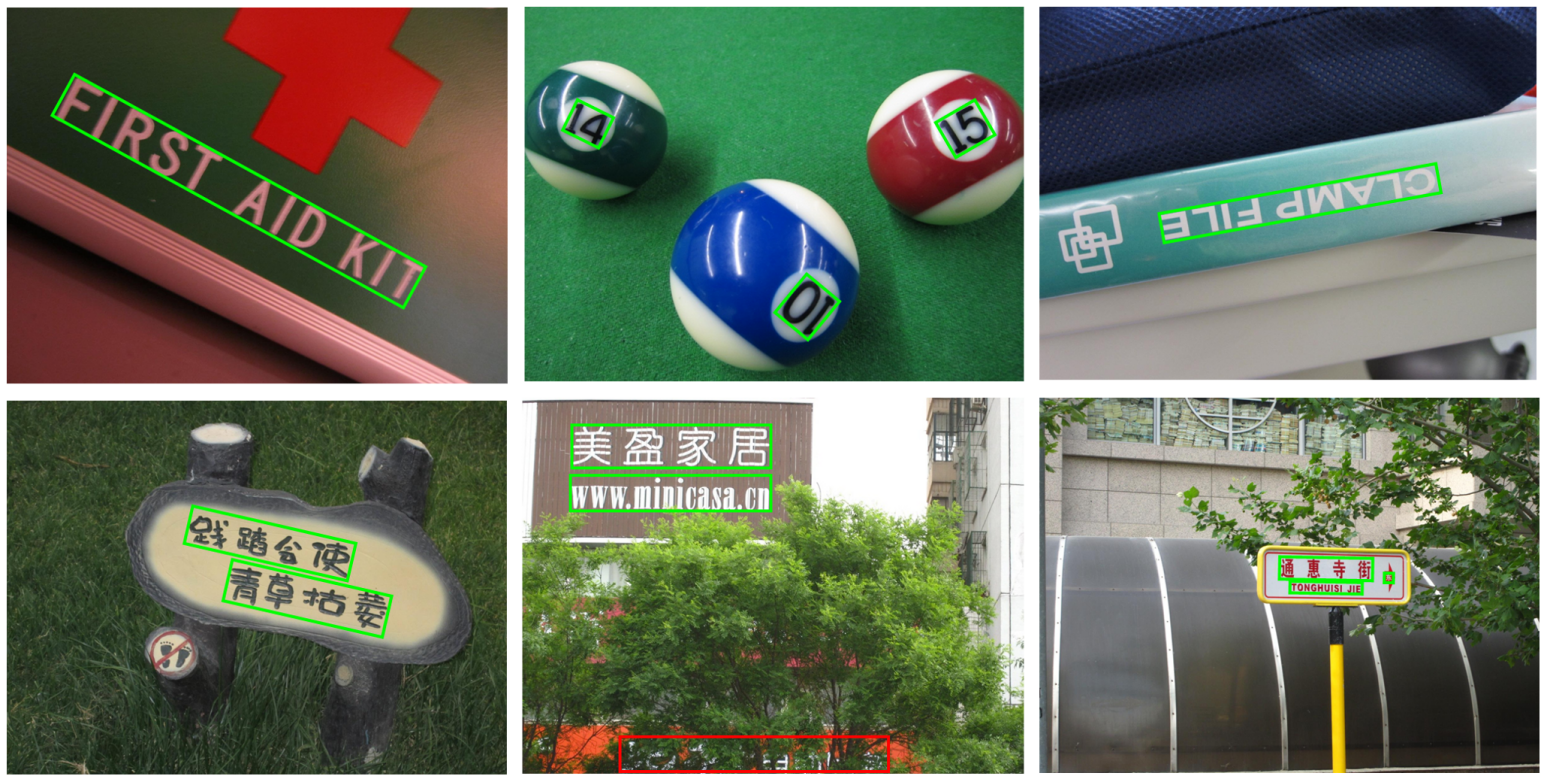
Typical images from the proposed dataset along with ground truth rectangles. Notice the red rectangles. They indicate the texts within them are labeled as difficult (due to blur or occlusion).

The next step of this stage is to group the pixels in the SWT image into connected components. The pixels are associated using a simple rule that the ratio of SWT values of neighboring pixels is less than 3.0. The connected components are shown in Fig. 3 (d) of [23]. Note the red rectangles in the image, where each rectangle contains a connected component.

In fact, the proposed pipeline is general and not specific to any kind of low level operator for component extraction. Though SWT is employed to extract components in this paper, other methods (such as MSER [11], [25]) that are able to reliably generate connected components corresponding to character candidates can also be used. We leave evaluation and comparison of different component extraction methods for future research.

#### Component analysis

The purpose of component analysis is to identify and eliminate the connected components that are unlikely parts of texts. To this end, we devise a two-layer filtering mechanism.

The first layer is a filter consists of a set of heuristic rules. This filter runs on a collection of statistical and geometric properties of components, which are very fast to compute. True text components usually have nearly constant stroke width and compact structure (not too thin and long), so width variation, aspect ratio and occupation ratio are chosen as the basic properties to filer out obvious non-text components.

For a connected component 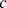 with 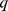 foreground pixels (black pixels in the SWT image), we first compute its bounding box 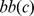 (its width and height are denoted by 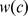 and 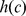, respectively) and the mean as well as standard deviation of the stroke widths, 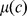 and 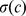. The basic properties are defined as follows:

- **Width variation.** Width variation measures the variation in stroke width of the component 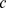: 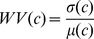.
- **Aspect ratio.** In horizontal conditions aspect ratio is defined as the ratio between the width and height of the component 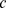. To accommodate texts of different directions, we use a new definition of aspect ratio: 
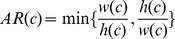
.
- **Occupation ratio.** Occupation ratio is used to remove non-text components caused by spurious rays in the SWT image. This property is defined as the ratio between the number of foreground pixels and area of the component 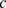: 
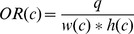
.

The valid ranges of these basic properties are empirically set to [0,1], [0.1,1] and [0.1,1], respectively. Components with one or more invalid properties will be taken as non-text regions and discarded. A large portion of obvious non-text components are eliminated after this step (notice the difference between Fig. 3 (d) of [23] and Fig. 3 (e) of [23]), suggesting that this preliminary filter is effective.

The second layer is a classifier trained to identify and reject the non-text components that are hard to remove with the preliminary filter. A collection of component level features, which capture the differences of geometric and textural properties between text components and non-text components, are used to train this classifier. The criteria for feature design are: scale invariance, rotation invariance and low computational cost. To meet these criteria, we propose to estimate the center, characteristic scale and major orientation of each component (Fig. 4 of [23]) before computing the component level features. Based on these characteristics, features that are both effective and computationally efficient can be obtained. The details of these component level features are discussed in Section **Component Level Features**.

**Figure 4:**
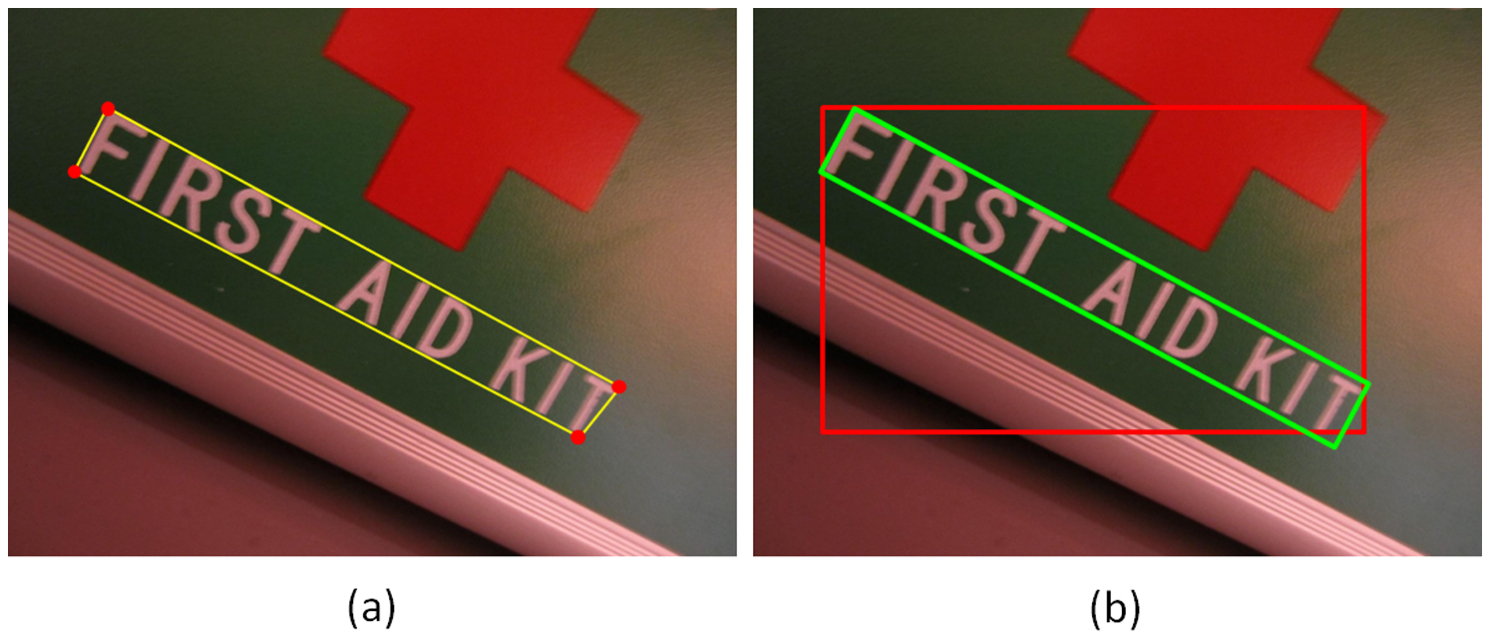
Ground truth generation. (a) Human annotation. The annotators are required to locate and bound each text line using a four-vertex polygon (red dots and yellow lines). (b) Ground truth rectangle (green). The ground truth rectangle is generated automatically by fitting a minimum area rectangle using the polygon.

For a component 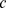, the barycenter 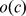, major axis 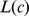, minor axis 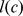, and orientation 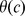 are estimated using Camshift [52] by taking the SWT image of component 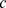 as distribution map. The center, characteristic scale and major orientation of component 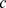 are defined as:
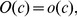
(1)

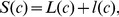
(2)

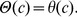
(3)


These characteristics are invariant to translation, scale and rotation to some degree (Fig. 4 of [23]). As we will explain in Section **Component Level Features**, this is the key to the scale and rotation invariance of the component level features.

We train a component level classifier using the component level features. Random Forest [53] is chosen as the strong classifier. The component level classifier is the first level of the two-level classification scheme. The probability of component 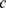, 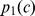, is the fraction of votes for the positive class (text) from the trees. The components whose probabilities are lower than a threshold  are eliminated and the remaining components are considered as character candidates (Fig. 3 (f) of [23]). To ensure high recall, the threshold 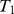 is set very low, as high threshold may filter out true text components.

#### Candidate linking

The character candidates are aggregated into chains at this stage. This stage also serves as a filtering step because the candidate characters cannot be linked into chains are taken as components accidentally formed by noises or background clutters, and thus are discarded.

Firstly, character candidates are linked into pairs. In [9], whether two candidates can be linked into a pair is determined based on the heights and widths of their bounding boxes. However, bounding boxes are not rotation invariant, so we use their characteristic scales instead. If two candidates have similar stroke widths (ratio between the mean stroke widths is less than 2), similar sizes (ratio between their characteristic scales does not exceed 2.5), similar colors and are close enough (distance between them is less than two times the sum of their characteristic scales), they are labeled as a pair. The above parameters are optimized using the training data of the ICDAR datasets [47]–[49], however, this parameter setting turns out to be effective for all the datasets used in this paper.

Unlike [9] and [11], which only consider horizontal or near-horizontal linkings, the proposed algorithm allows linkings of arbitrary directions. This endows the system with the ability of detecting multi-oriented texts, not limited to horizontal texts.

Next, a greedy hierarchical agglomerative clustering [54] method is applied to aggregate the pairs into candidate chains. Initially, each pair constitutes a chain. Then the similarity between each couple of chains that share at least one common candidate and have similar orientations is computed; chains with the highest similarity are merged together to form a new chain. The orientation consistency 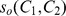 and population consistency 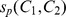 between two chains 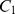 and 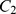, which share at least one common candidate, are defined as:
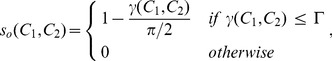
(4)and
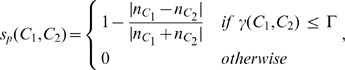
(5)where 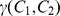 is the included angle of 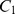 and 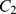 while 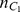 and 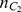 are the candidate numbers of 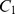 and 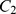. 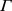 is used to judge whether two chains have similar orientations and is empirically set to 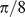. The similarity between two chains 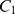 and 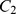 is defined as the harmonic mean [55] of their orientation consistency and population consistency:



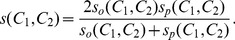
(6)According to this similarity definition, the chains with proximal sizes and orientations are merged with priority. This merging process proceeds until no chains can be merged.

At last, the character candidates not belonging to any chain are discarded. The candidate chains after aggregation are shown in Fig. 3 (g) of [23], in which each green line represents a chain.

#### Chain analysis

The candidate chains formed at the previous stage might include false positives that are random combinations of scattered background clutters (such as leaves and grasses) and repeated patterns (such as bricks and windows). To eliminate these false positives, a chain level classifier is trained using the chain level features.

Random Forest [53] is again used. The chain level classifier is the second level of the two-level classification scheme. The probability of chain 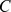, 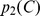, is the fraction of votes for the positive class (text) from the trees. The chains with probabilities lower than a threshold 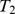 are eliminated.

To make better decisions, the total probability of each chain is also calculated. For a chain 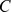 with 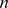 candidates 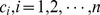, the total probability is defined as:
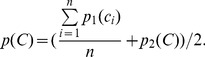
(7)


The chains whose total probabilities are lower than a threshold 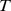 are discarded.

As texts of different orientations are considered, the remaining chains may be in any direction. Therefore, a candidate might belong to multiple chains. For example, in Fig. 3 (h) of [23] the character ‘ P’ in the first line is linked in three chains (note the green lines). In reality, however, a character is unlikely to belong to multiple text lines. If several chains compete for the same candidate, only the chain with the highest total probability will survive (note the difference between Fig. 3 (h) and (i) in [23]).

The survived chains are outputted by the system as detected texts (Fig. 3 (j) of [23]). For each detected text, its orientation is calculated through linear least squares [54] using the centers of the characters; its minimum area rectangle [56] is estimated using the orientation and the bounding boxes of the characters. Word partition, which divides text lines into separate words, is also implemented in the proposed algorithm; but it is not shown, since the general task of text detection does not require this step.

The whole algorithm described above is performed twice to handle both bright text on dark background and dark text on bright background, once along the gradient direction and once along the inverse direction. The results of two passes are fused to make final decisions. For clarity, only the results of one pass are presented.

### Feature Design

We design two collections of features, component level features and chain level features, for classifying text and non-text, based on the observation that it is the median degree of regularities of text rather than particular color or shape that distinguish it from non-text, which usually has either low degree (random clutters) or high degree (repeated patterns) of regularities. At character level, the regularities of text come from nearly constant width and texturelessness of strokes, and piecewise smoothness of stroke boundaries; at line level, the regularities of text are similar colors, sizes, orientations and structures of characters, and nearly constant spacing between consecutive characters.

#### Component level features

Inspired by Shape Context [57] and Feature Context [58], we devise two templates (Fig. 5 (a) of [23]) to capture the regularities of each component in coarse and fine granularity, respectively. The radius and orientation of the templates are not stationary, but adaptive to the component. When computing descriptors for a component, each template is placed at the center and rotated to align with the major orientation of the component; the radius is set to the characteristic scale of the component. Different cues from the sectors are encoded and concatenated into histograms. In this paper, the following cues are considered for each sector:

**Figure 5:**
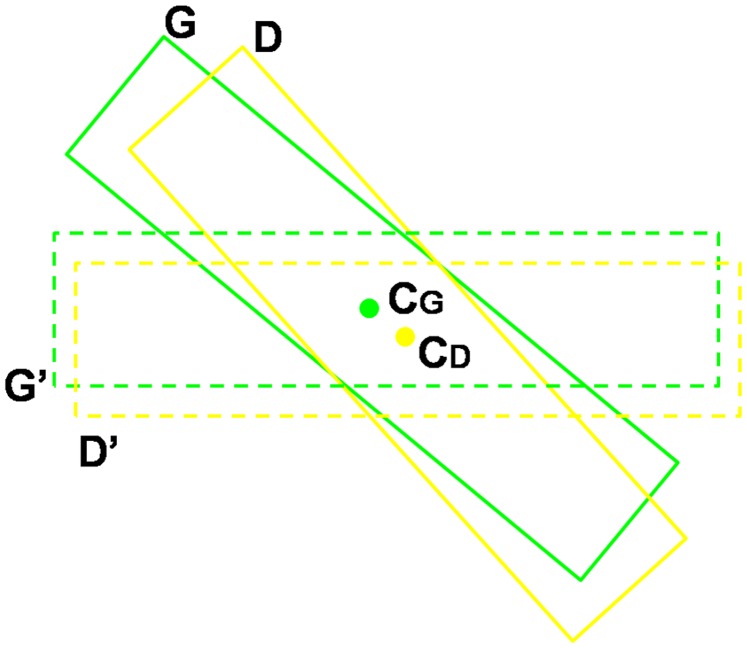
Calculation of overlap ratio between detection rectangle and ground truth rectangle.

- **Contour shape** [**59**] **.** Contour shape is a histogram of oriented gradients. The gradients are computed on the component contour (Fig. 5 (c) of [23]).
- **Edge shape** [**59**] **.** Edge shape is also a histogram of oriented gradients; but the gradients are computed at all the pixels in the sector (Fig. 5 (d) of [23]).
- **Occupation ratio.** Occupation ratio is defined as the ratio between the number of the foreground pixels of the component within the sector and the sector area (Fig. 5 (e) of [23]).

To achieve rotation invariance, the gradient orientations are rotated by an angle 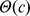, before computing contour shape and edge shape. Then, the gradient orientations are normalized to the range 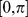. Six orientation bins are used for computing histograms of contour shape and edge shape, to cope with different fonts and local deformations.

For each cue, the signals computed in all the sectors of all the templates are concatenated to form a descriptor. We call these descriptors scalable rotative descriptors, because they are computed based on templates that are scalable and rotative. Scalable rotative descriptors are similar to PHOG [60], as they both adopt spatial pyramid representation [61]. Different from the templates used for computing PHOG, our templates are circular and their scale and orientation are adaptive to the component being described. This is the key to the scale and rotation invariance of these descriptors.

It is widely accepted in the community that alignment is very important for recognition and classification tasks [62], [63], as it can moderate or even eliminate the negative effects caused by transformations and thus lead to more robust measurement and similarity. Our strategy for computing scalable rotative descriptors, i.e. estimating center, characteristic scale and major orientation of components and calculating features using adaptive templates, is actually a kind of implicit alignment of components. This strategy can be generalized to multi-oriented text recognition either.

The characteristic scale is crucial for the computation of scalable rotative descriptors, because it directly determines the scales of the templates. Too small templates may miss important information of components while too large templates may introduce noises and interferences from other components and background. The value of characteristic scale calculated using Eqn. 2 is a good trade-off in practice.

We have found through experiments (not shown in this paper) that using finer templates can slightly improve the performance, but will largely increase the computational burden.

In addition, another three types of rotation and scale invariant features are considered:

- **Axial ratio.** Axial ratio is computed by dividing the major axis of the component 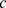 with its minor axis: 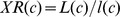.
- **Width variation.** This feature is the same as defined in Sec. **Component Analysis**.
- **Density.** The density of component 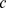 is defined as the ratio between its pixel number 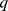 and characteristic area (here the characteristic area is 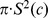, not the area of the bounding box): 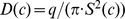.

#### Chain level features

Eleven types of chain level features, which are not specific to rotation and scale, are designed to discriminate text lines from false positives (mostly repeated patterns and random clutters) that cannot be distinguished by the component level features.

For a candidate chain 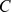 with 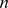 (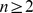) candidates 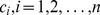, the features are defined as below and summarized in Tab. 1:

**Table 1:** Chain level features.

Feature	Definition
Candidate count	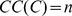
Average probability	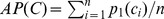
Average turning angle	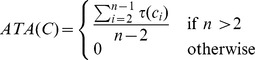
Size variation	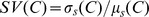 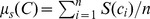 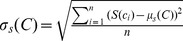
Distance variation	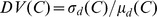 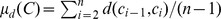 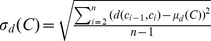
Average direction bias	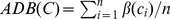
Average axial ratio	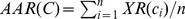
Average density	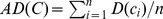
Average width variation	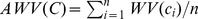
Average color self-similarity	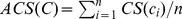 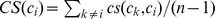
Average structure self-similarity	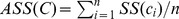 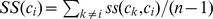

- **Candidate count.** This feature is adopted based on the observation that false positives usually have very few (for random clutters) or too many (for repeated patterns) candidates.
- **Average probability.** The probabilities given by the component level classifier are reliable. This feature is the average of all the probabilities (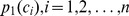) of the candidates belonging to 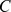.
- **Average turning angle.** Most texts present in linear form, so for a text line the mean of the turning angles at the interior characters (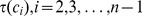) is very small; however, for random clutters this property will not hold. 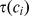 is the included angle between the line 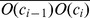 and 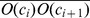.
- **Size variation.** In most cases characters in a text line have approximately equal sizes; but it’s not that case for random clutters. The size of each component is measured by its characteristic scale 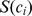.
- **Distance variation.** Another property of text is that characters in a text line are distributed uniformly, i.e. the distances between consecutive characters have small deviation. The distance between two consecutive components is the distance of their centers 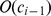 and 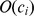.
- **Average direction bias.** For most text lines, the major orientations of the characters are nearly perpendicular to the major orientation of the text line. Direction bias of component 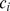, 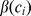, is the included angle between 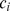 and the orientation of the chain.
- **Average axial ratio.** Some repeated patterns (e.g. barriers) that are not texts consist of long and thin components, this feature can help differentiate them from true texts.
- **Average density.** On the contrary, other repeated patterns (e.g. bricks) consist of short and fat components, this feature can be used to eliminate this kind of false positives.
- **Average width variation.** False positives formed by foliage usually have varying widths while texts have constant widths. This feature is defined as the mean of all the width variation values of the candidates.
- **Average color self-similarity.** Characters in a text line usually have similar but not identical color distributions with each other; yet in false positive chains, color self-similarities [64] of the candidates are either too high (repeated patterns) or too low (random clutters). The color similarity 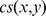 is defined as the cosine similarity of the color histograms of the two candidates 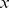 and 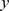.
- **Average structure self-similarity.** Likewise, characters in a text line have similar structure with each other while false positives usually have almost the same structure (repeated patterns) or diverse structures (random clutters). The structure similarity 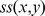 is defined as the cosine similarity of the edge shape descriptors of the two components 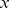 and 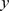.

## Dataset and Evaluation Protocol

In this section, we introduce a large dataset for evaluating text detection algorithms, which contains 500 natural images with real-world complexity. In addition, a new evaluation methodology which is suitable for benchmarking algorithms designed for texts of arbitrary directions is proposed.

### Dataset

Although widely used in the community, the ICDAR datasets [47]–[49] have two major drawbacks. First, most of the text lines (or single characters) in the ICDAR datasets are horizontal. In real scenarios, however, text may appear in any orientation. The second drawback is that all the text lines or characters in this dataset are in English. Therefore it is unable to use these datasets to assess detection systems designed for multilingual scripts.

These two shortcomings have been pointed out in [13], [14]. Two separate datasets are therefore created: one contains non-horizontal text lines [14] and the other one is a multilingual dataset [13]. In this work, we generate a new multilingual text image dataset with horizontal as well as slant and skewed texts. We name this dataset MSRA Text Detection 500 Database (MSRA-TD500), because it includes 500 natural images in total. These images are taken from indoor (office and mall) and outdoor (street) scenes using a packet camera. The indoor images are mainly signs, doorplates and caution plates while the outdoor images are mostly guide boards and billboards in complex background. The resolutions of the images vary from 1296×864 to 1920×1280. This dataset is available at http://www.loni.ucla.edu/~ztu/publication/MSRA-TD500.zip.

MSRA-TD500 is very challenging because of both the diversity of the texts and the complexity of the backgrounds in the images. The texts may be in different languages (Chinese, English and mixture of both), fonts, sizes, colors and orientations. The backgrounds may contain vegetation (e.g. trees and grasses) and repeated patterns (e.g. windows and bricks), which are not so distinguishable from text.

Some typical images from this dataset are shown in Fig. 3. It is worth mentioning that even though the purpose of this dataset is to evaluate text detection algorithms designed for multi-oriented texts, horizontal and near-horizontal texts still dominate the dataset (about 2/3) because these are the most common cases in practice.

The dataset is divided into two parts: training set and test set. The training set contains 300 images randomly selected from the original dataset and the rest 200 images constitute the test set. All the images in this dataset are fully annotated. The basic unit in this dataset is text line rather than word, which is used in the ICDAR dataset, because it is hard to partition Chinese text lines into individual words based on their spacings; even for English text lines, it is non-trivial to perform word partition without high level information. The procedure of ground truth generation is shown in Fig. 4.

### Evaluation Protocol

Before presenting our novel evaluation protocol for text detection, we first introduce the evaluation method used in the ICDAR competitions [47], [48] as background. Under the ICDAR evaluation protocol, the performance of an algorithm is measured by F-measure, which is the harmonic mean of precision and recall. Different from the standard information retrieval measures of precision and recall, more flexible definitions are adopted in the ICDAR competitions [47], [48]. The match 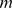 between two rectangles is defined as the ratio of the area of intersection and that of the minimum bounding rectangle containing both rectangles. The set of rectangles estimated by each algorithm are called *estimates* while the set of ground truth rectangles provided in the ICDAR dataset are called *targets*. For each rectangle, the match with the largest value is found. Hence, the best match for a rectangle 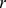 in a set of rectangles 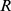 is:
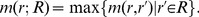
(8)


Then, the definitions of precision and recall are:
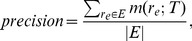
(9)

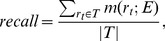
(10)where 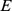 and 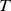 are the sets of ground truth rectangles and estimated rectangles, respectively. The F-measure, which is a single measure of algorithm performance, is a combination of the two above measures. The relative weights of precision and recall are controlled by a parameter 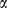, which is set to 0.5 to give equal weights to precision and recall:



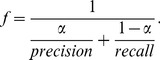
(11)Minimum area rectangles [56] are used in our protocol because they (green rectangle in Fig. 4 (b)) are much tighter and more accurate than axis-aligned rectangles (red rectangle in Fig. 4 (b)). However, a problem imposed by using minimum area rectangles is that it is difficult to judge whether a text line is correctly detected. As shown in Fig. 5, it is not trivial to directly compute the overlap ratio between the estimated rectangle 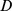 and the ground truth rectangle 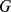. Instead, we calculate the overlap ratio using axis-aligned rectangles 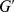 and 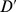, which are obtained by rotating 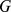 and 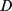 round their centers 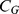 and 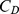, respectively. The overlap ratio between 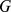 and 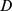 is defined as:
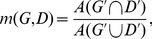
(12)where 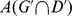 and 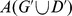 denote the areas of the intersection and union of 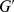 and 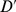. Obviously, the overlap ratio computed in this way is not accurate. Besides, the ground truth rectangles annotated are not accurate either, especially when the texts are skewed. Because of the imprecision of both ground truth and computed overlap ratio, the definitions of precision and recall used in the ICDAR protocol do not apply. Alternatively, we return to their original definitions.

Similar to the evaluation method for the PASCAL object detection task [65], in our protocol detections are considered true or false positives based on the overlap ratio between the estimated minimum area rectangles and the ground truth rectangles. If the included angle of the estimated rectangle and the ground truth rectangle is less than 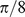 and their overlap ratio exceeds 0.5, the estimated rectangle is considered a correct detection. Multiple detections of the same text line are taken as false positives. The definitions of precision and recall are:
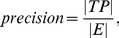
(13)

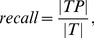
(14)where 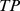 is the set of true positive detections while 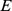 and 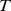 are the sets of estimated rectangles and ground truth rectangles.

Moreover, to accommodate difficult texts (too small, occluded, blurry, or truncated) that are hard for text detection algorithms, we introduce an elastic mechanism which can tolerate detection misses of difficult texts. The basic criterion of this elastic mechanism is: *if the difficult texts are detected by an algorithm, it counts; otherwise, the algorithm will not be punished*. Accordingly, the annotations of the images in the proposed dataset should be changed. Each text line considered to be difficult is given an additional “ difficult” label (Fig. 3). Thus the ground truth rectangles can be categorized into two sub sets: ordinary sub set 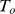 and difficult sub set 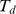; ditto, the true positives 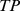 can also be categorized into ordinary sub set 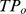, which is the set of rectangles matched with 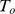, and ordinary sub set 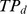, which is the set of rectangles matched with 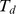. After incorporating the elastic mechanism, the definitions of precision and recall become:
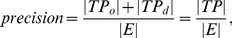
(15)

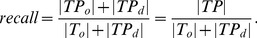
(16)


## Experiments and Discussions

We have implemented the proposed algorithm in C++ and have evaluated it on a common server (2.53 GHz CPU, 48G RAM and Windows 64-bit OS). 200 trees are used for training the component level classifier and 100 trees for the chain level classifier. The threshold values are: 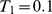, 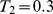 and 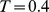. We have found empirically that the text detectors under this parameter setting work well for all the datasets used in this paper.

### Results on Horizontal Texts

In order to compare the proposed algorithm with existing methods designed for horizontal texts, we have evaluated the algorithm on the standard dataset used in the ICDAR 2003 Rubust Reading Competition [47] and the ICDAR 2005 Text Locating Competition [48]. This dataset contains 509 fully annotated text images. 258 images from the dataset are used for training and 251 for testing. We train a text detector (denoted by TD-ICDAR) on the training images.

Some detected texts of the proposed algorithm are presented in Fig. 7 of [23]. Our algorithm can handle several types of challenges, e.g. variations in text font, color and size, as well as repeated patterns and background clutters. The quantitative comparison of different methods evaluated on the ICDAR test set is shown in Tab. 2 of [23]. As can be seen, our method compares favorably with the state-of-the-art when dealing with horizontal texts.

**Table 2:** Performances of different text detection methods evaluated on the ICDAR 2011 dataset [49].

Algorithm	Precision	Recall	F-measure
TD-ICDAR2011	0.7215	0.5952	0.6523
Kim *et al.* [49]	**0.8298**	**0.6247**	**0.7128**
Yi *et al.* [49]	0.6722	0.5809	0.6232
Yang *et al.* [49]	0.6697	0.5768	0.6198
Neumann *et al.* [49]	0.6893	0.5254	0.5963
Shao *et al.* [49]	0.6352	0.5352	0.5809
Guyomard *et al.* [49]	0.6297	0.5007	0.5578
Lee *et al.* [49]	0.5967	0.4457	0.5103
Sun *et al.* [49]	0.3501	0.3832	0.3659
Hanif *et al.* [49]	0.5505	0.2596	0.3419

It is noted that existing algorithms seem to converge in performance (with F-measure around 0.66) on the ICDAR dataset. This might be due to three reasons: (1) the ICDAR evaluation method is different from the conventional methods for object detection (e.g. the PASCAL evaluation method [65]). The ICDAR evaluation method actually requires pixel-level accuracy (see Eqn. 9 and Eqn. 10), which is rigorous for detection algorithms, considering that the ground truth is given in the form of rough rectangles. (2) The ICDAR evaluation method requires word partition, that is, dividing text lines into individual words. This limits the scores of text detection algorithms either; because it is non-trivial to perform word partition without high level information. Moreover, the definitions of “word” are not consistent among different images. (3) Most algorithms assume that in the image a word or text line consists of at least two characters. However, in the ICDAR dataset some images contain single characters. In these images, most existing algorithms will fail to detect the single characters.

The ICDAR 2011 Robust Reading Competition Challenge 2 [49] was held to track the recent progress in the filed of scene text detection and recognition. Due to the problems with the dataset used in the previous ICDAR competitions (for example, imprecise bounding boxes and inconsistent definitions of “word”), the dataset in the ICDAR 2011 competition is extended and relabeled [49]. Moreover, the evaluation method proposed by Wolf *et al.* [66] is adopted as the standard for performance evaluation, to replace the previous evaluation protocol, which is unable to handle the cases of one-to-many and many-to-many matches and thus consistently underestimates the capability of text detection algorithms.

**Figure 6:**
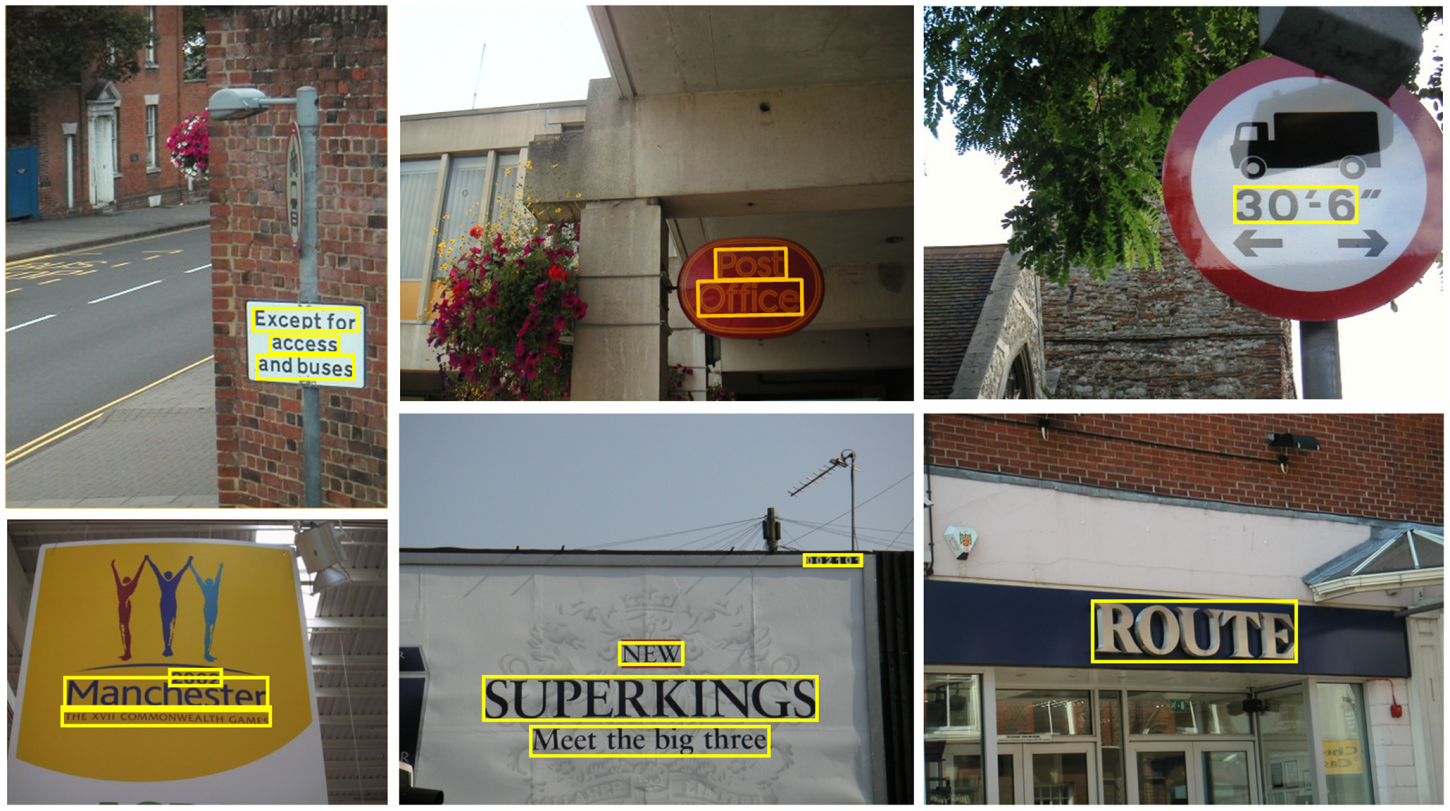
Detected texts in images from the ICDAR 2011 test set.

To enable fair comparison, we have also trained a text detector (denoted by TD-ICDAR2011) using the training set of the ICDAR 2011 competition dataset, performed text detection on the test images and measured the performance using the method of Wolf *et al.* [66]. Fig. 6 illustrates several detection examples of our method on this dataset. The quantitative results of different text detection methods on the ICDAR 2011 dataset are shown in Tab. 2. The proposed algorithm achieves the second highest F-measure on this dataset.

### Results on Multi-oriented Texts

We have also trained a text detector (denoted by TD-MSRA) on mixture of the training set of the proposed dataset and that of ICDAR and compared it to the systems of Epshtein *et al.* [9] and Chen *et al.* [7]. The executables of these system are obtained from the authors. Detection examples of the proposed algorithm on this dataset are shown in Fig. 8 (a) of [23]. The proposed algorithm is able to detect texts of large variation in natural scenes, e.g., skewed and curved text. The images in the last row of Fig. 8 (a) of [23] are some typical cases where our algorithm failed to detect the texts or gave false positives. The misses (pink rectangles) are mainly due to strong highlights, blur and low resolution; the false positives (red rectangles) are usually caused by elements that are very alike text, such as windows, trees, and signs.

The performances are measured using the proposed evaluation protocol and shown in Tab. 3 of [23]. Compared with the competing algorithms, the proposed method achieves significantly enhanced performance when detecting texts of different orientations. The performances of other competing algorithms are not presented because of unavailability of their codes/executables. The average processing time of our algorithm on this dataset is 7.2 s and that of Epshtein *et al.* is 6 s. Our algorithm is a bit slower, but with the advantage of being able to detect multi-oriented texts.

**Table 3:** Performances of different text detection methods evaluated on texts of different languages.

Algorithm	Precision	Recall	F-measure
TD-MSRA	0.73	0.64	0.66
Epshtein *et al.* [9]	0.58	0.65	0.59
Chen *et al.* [7]	0.06	0.08	0.07

In [14], a dataset called Oriented Scene Text Database (OSTD), which contains texts of various orientations, is released. This dataset includes 89 images of logos, indoor scenes and street views. We perform text detection on all the images in this dataset. The quantitative results are presented in Tab. 4 of [23]. Our method outperforms [14] on the Oriented Scene Text Database (OSTD), with an improvement of 0.17 in F-measure.

**Table 4:** End-to-end scene text recognition performances.

System	Precision	Recall	F-measure
Ours	0.58	0.51	0.53
Epshtein *et al.* [9]	0.57	0.49	0.51
Direct OCR	0.13	0.10	0.11

From Tab. 3 and 4 of [23], we observe that even TD-ICDAR (only trained on horizontal texts) achieves much better performance than other methods on non-horizontal texts. It demonstrates the effectiveness of the proposed rotation-invariant features.

### Results on Texts of Different Languages

**Figure 7:**
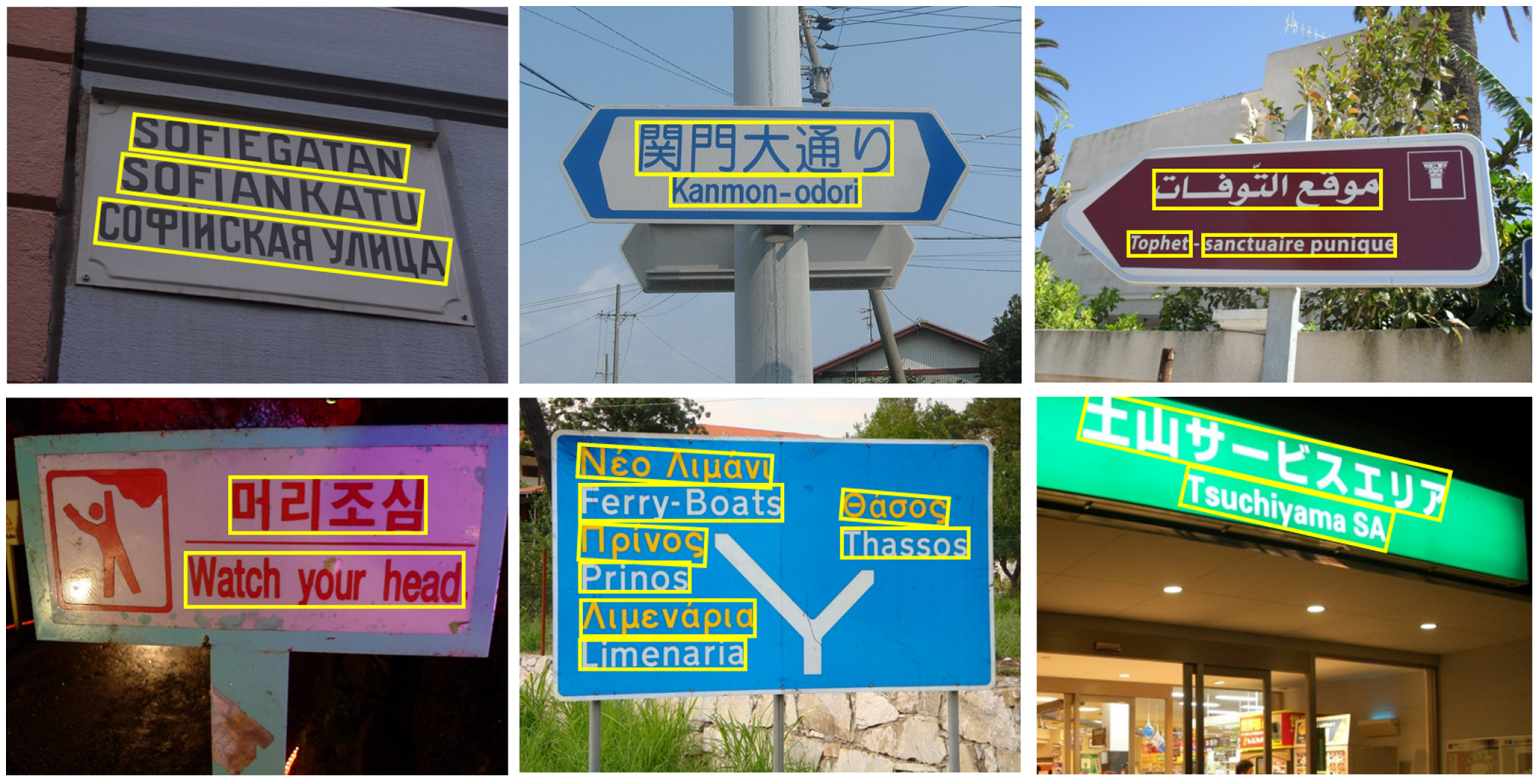
Detected texts in various languages. The images are collected from the Internet.

To further verify the ability of the proposed algorithm to detect texts of different languages, we have collected a multilingual text image database (Will be available at http://www.loni.ucla.edu/~ztu/publication/) from the Internet. The database contains 94 natural images with texts of various languages, including both oriental and western languages, such as Japanese, Korean, Arabic, Greek, and Russian. We apply TD-MSRA to all the images in this database. Fig. 7 shows some detected texts in images from this database. The algorithms of Epshtein *et al.* [9] and Chen *et al.* [7] are adopted as baselines. The quantitative results of these algorithms are presented in Tab. 3. The proposed algorithm and the method of Epshtein *et al.* [9] both give excellent performance on this benchmark. Though TD-MSRA is only trained on Chinese and English texts, it can effortlessly generalize to texts in different languages. This indicates that the proposed algorithm is quite general and it can serve as a multilingual text detector.

### Special Consideration on Single Characters

**Figure 8:**
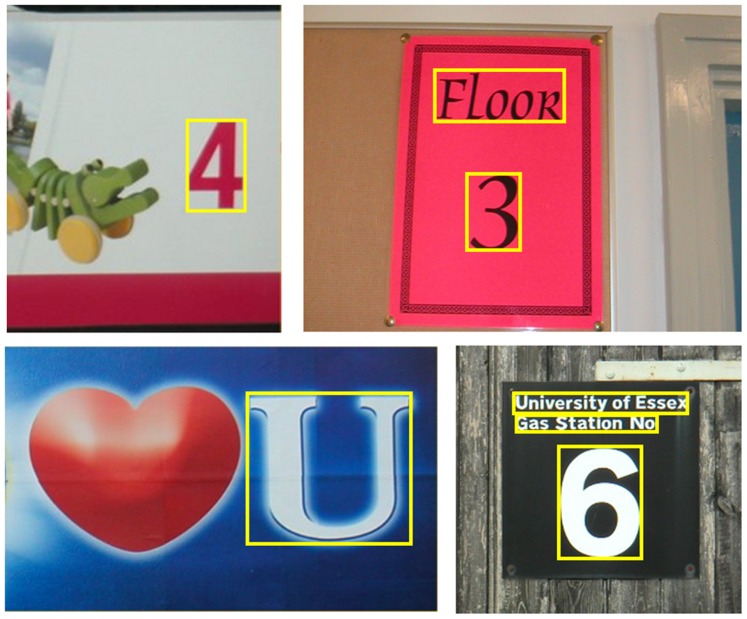
Detected single characters in images. Images are from the ICDAR dataset [47], [48].

Most existing algorithms cannot handle single characters, since they assume that in the image a word or text line consists of at least two characters. To overcome this limitation, we have modified the proposed algorithm to handle single characters. In the candidate linking stage, we no longer simply discard all single character candidates but instead retain the character candidates with high probabilities (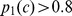), even if they do not belong to any chain. After this modification, the proposed algorithm is able to detect obvious single characters in natural images. Fig. 8 depicts some detected single characters by the proposed algorithm.

To assess the effectiveness of the proposed strategy for single character detection, we have conducted an additional experiment. The algorithm is applied to the images containing single characters from the ICDAR dataset [47], [48], with and without single character detection. Without single character detection, the algorithm achieves precision = 0.56, recall = 0.28 and F-measure = 0.36; with single character detection, the algorithm achieves precision = 0.62, recall = 0.40 and F-measure = 0.47. The performance is significantly improved after enabling single character detection.

### End-to-End Scene Text Recognition

**Figure 9:**
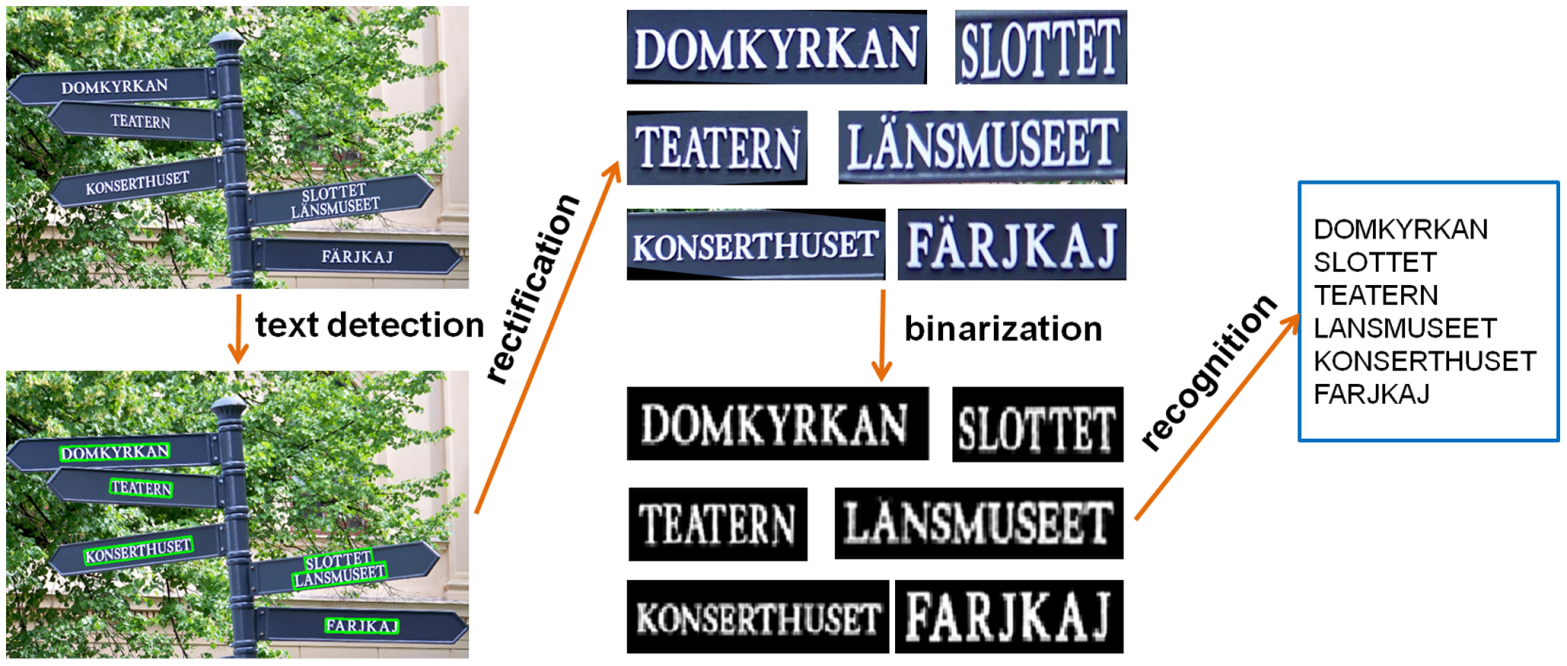
Pipeline of our end-to-end scene text recognition system.

As can be seen from previous experiments, the proposed text detection algorithm works very well under fairly broad realistic conditions. Thus, one could combine it with any of the existing Optical Character Recognition (OCR) engines to build an end-to-end recognition system for multi-oriented text. A likely pipeline of such a system is illustrated in Fig. 9: We first apply our text detection algorithm to the original image. If the detected text regions have significant deformation, we then rectify them by the low-rank structure based rectification technique TILT [67]. Next, we binarize the text regions with adaptive thresholding and feed the binary images into an off-the-shelf OCR software [68] to produce the final recognition result.

**Figure 10:**
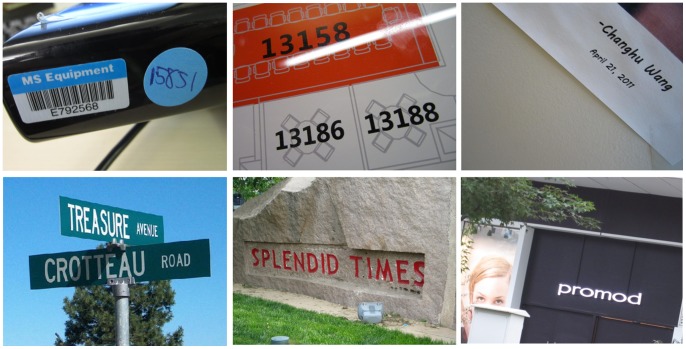
Examples of the images collected for end-to-end scene text recognition.

Since there is no standard benchmark for multi-oriented English text recognition (The NEOCR dataset [69] includes images with multi-oriented texts in natural scenes. However, the texts in this database are in different languages, such as Hungarian, Russian, Turkish and Czech, which are not supported by our end-to-end recognition system currently), we collect a dataset (Will be available at http://www.loni.ucla.edu/~ztu/publication/) of 80 natural images with slant and skewed English texts and Arabic numbers, to evaluate the proposed end-to-end text recognition system. Majority of the images are from the MSRA-TD500 database and the rest images are from the Internet. Fig. 10 shows several typical images from this database.

For comparison, we have tested the end-to-end text recognition system of Epshtein *et al.* [9] on this dataset. To demonstrate how text detection can help effectively extract text information from natural images, we have also performed character recognition directly on the original images (denoted by Direct OCR). The quantitative performances are computed at character level and shown in Tab. 4. As can be seen, applying OCR directly to natural images gives very poor performance, because of the variations of texts and background clutters. In contrast, both our scene text recognition system and that of Epshtein *et al.* [9] achieve much higher performance. This suggests that text detection is a crucial step when extracting text information from natural images.

We have also examined why in this experiment the improvement of our system over that of Epshtein’s is not so dramatic as in previous pure detection experiments. The main reason is that although our system can detect more texts, some of the fonts in these natural images cannot be handled well by the current OCR system. This suggests that to build a truly high-performance text recognition system for texts in natural images, there is still significant challenge for further improvement in the text recognition component, especially in recognizing texts with more diverse fonts, sizes, and orientations. From our observation and preliminary study, some of the discriminative features that we have extracted for detection purpose can be very useful for subsequent text recognition as well. We leave a more careful study of a unified text detection and recognition system for future work.

### Conclusions

We have presented a text detection system that is capable of detecting texts of varying directions in complex natural scenes. Our system compares favorably with the state-of-the-art algorithms when handling horizontal texts and achieves significantly enhanced performance on multi-oriented texts. Furthermore, we have proposed a multilingual database with horizontal as well as non-horizontal texts and specifically designed an evaluation protocol for benchmarking algorithms for multi-oriented texts.

The component level features are actually character descriptors that can distinguish among different characters, thus they can be adopted to recognize characters. We plan to make use of this property and develop a unified framework for text detection and character recognition in the future.
